# PIEZO1 targeting in macrophages boosts phagocytic activity and foam cell apoptosis in atherosclerosis

**DOI:** 10.1007/s00018-024-05372-3

**Published:** 2024-08-06

**Authors:** Shirin Pourteymour, Jingxue Fan, Rakesh Kumar Majhi, Shuyuan Guo, Xin Sun, Zhen Huang, Ying Liu, Hanna Winter, Alexandra Bäcklund, Nikolaos-Taxiarchis Skenteris, Ekaterina Chernogubova, Olivera Werngren, Zhaolong Li, Josefin Skogsberg, Yuhuang Li, Ljubica Matic, Ulf Hedin, Lars Maegdefessel, Ewa Ehrenborg, Ye Tian, Hong Jin

**Affiliations:** 1 Department of Medicine (Solna), Karolinska Institutet, Stockholm, Sweden; 2 Department of Nutrition, Institute of Basic Medical Sciences, Faculty of Medicine, University of Oslo, Blindern, PO Box 1046, 0317 Oslo, Norway; 3 Department of Cardiology, The First Affiliated Hospital, Cardiovascular Institute, Harbin Medical University, Harbin, People’s Republic of China; 4 Department of Microbiology, Tumor and Cell Biology, Division of Clinical Microbiology, Karolinska Institutet, Stockholm, Sweden; 5 Department of Cardiology, Shenzhen Cardiovascular Minimally Invasive Medical Engineering Technology Research and Development Center, Shenzhen People’s Hospital, Shenzhen, People’s Republic of China; 6 Department of Vascular and Endovascular Surgery, Technical University Munich, Munich, Germany; 7 Department of Molecular Medicine and Surgery, Karolinska Institutet, Stockholm, Sweden; 8 Department of Medical Biochemistry and Biophysics, Karolinska Institutet, Stockholm, Sweden

**Keywords:** PIEZO1, Macrophages, Atherosclerosis, Mitochondria, Reactive oxygen species

## Abstract

The rising incidences of atherosclerosis have necessitated efforts to identify novel targets for therapeutic interventions. In the present study, we observed increased expression of the mechanosensitive calcium channel Piezo1 transcript in mouse and human atherosclerotic plaques, correlating with infiltration of PIEZO1-expressing macrophages. In vitro administration of Yoda1, a specific agonist for PIEZO1, led to increased foam cell apoptosis and enhanced phagocytosis by macrophages. Mechanistically, PIEZO1 activation resulted in intracellular F-actin rearrangement, elevated mitochondrial ROS levels and induction of mitochondrial fragmentation upon PIEZO1 activation, as well as increased expression of anti-inflammatory genes. In vivo, ApoE^−/−^ mice treated with Yoda1 exhibited regression of atherosclerosis, enhanced stability of advanced lesions, reduced plaque size and necrotic core, increased collagen content, and reduced expression levels of inflammatory markers. Our findings propose PIEZO1 as a novel and potential therapeutic target in atherosclerosis.

**Supplementary Information:**

The online version contains supplementary material available at 10.1007/s00018-024-05372-3.

## Introduction

Arterial inflammation and dysregulated uptake of oxidized-LDL by infiltrating monocyte-derived macrophages and vascular smooth muscle cells in the arterial wall manifests as atherosclerosis [1–4]. Subsequently, excessive cholesterol uptake beyond the homeostatic capacity of macrophages and vascular smooth muscle cells impairs their lipid metabolism and transforms them to lipid-laden foam cells [5, 6]. After undergoing apoptosis, these foam cells experience a lingered imbalance in the clearance of apoptotic cells by phagocytic macrophages. Subsequent post-apoptotic necrosis and the release of toxic and pro-inflammatory contents result in the persistence of inflammation, enlargement of the necrotic core, and instability in the plaque [7, 8]. Furthermore, it has recently been suggested that an impaired “eat me signal” in foam cells and compromised efferocytosis by macrophages contributes to pathological complications [9].

In addition, proliferation of arterial resident macrophages and trans-differentiation of smooth muscle cells to macrophage-like cells may also contribute to lesion pathogenesis [4, 10, 11]. To this end, enhanced clearance of apoptotic cells from atherosclerotic lesions by macrophages signifies a promising field for potential therapeutic intervention.

In addition to the well-known genes associated with smooth muscle cells proliferation and foam cells formation, the roles of ion channels in atherosclerosis, which can be potentially regulated with pharmacological treatments are less elucidated. Therefore, in the present study, we initially investigated the gene expression pattern of key membrane ion channels that regulate cell mobility and function in murine models to identify the most dysregulated ion channel and its potential role in atherosclerosis. Notably, among the significantly altered seven ion channel genes in our transcriptome array data, Piezo-type mechanosensitive ion channel component 1 (Piezo1), emerged as the most upregulated gene. PIEZO1 acts as a mechanosensitive ion channel in human myocardiocytes [12], red blood cells [13], circulating immune cells [14, 15] and cardiac fibroblasts [16]. Importantly, various functional roles of PIEZO1 in the cardiovascular system have been reported such as sensing blood flow, regulation of vascular endothelial and smooth muscle responses, and controlling blood pressure [17–20]. Piezo1 deficient mice die during mid-gestation, primarily due to defects in cardiovascular development [18]. There is paucity of information on the role of PIEZO1 in macrophages and foam cells, especially in the context of atherosclerosis.

Hence, we have explored the functional role of PIEZO1 in macrophages through in vitro and in vivo experiments to harness the therapeutic potential of PIEZO1 in the context of atherosclerosis.

## Materials and methods

### Mouse transcriptome array analysis

Two separate mouse atherosclerosis whole transcriptome array databases (MTA) were used [21, 22]. Firstly, a mouse model of atherosclerotic lesion formation induced by incomplete ligation of the common carotid artery and conical polyethylene cuff implantation was used on 12 weeks old male ApoE Knockout mice. 4 weeks after ligation with additional 4 days after cuff implantation, carotid arteries were harvested for RNA isolation and array analysis [21]. Secondly, a section of the aorta from the aortic root to the third rib was harvested every week from *Ldlr*^−/−^ *Apo*100/100 Mttp^flox/flox^ Mx1-Cre mice with a plasma lipoprotein profile similar to that of familial hypercholesterolemia (Ldlr2/2Apob100/100), which were fed on chow diet containing 4% fat for 60 weeks (GSE38574).

### Human carotid artery plaque preparation for histology and RT-qPCR

Human carotid atherosclerotic plaques were from patients undergoing carotid endarterectomy and stored in Munich Vascular Biobank [23]. Control arteries were derived from deceased organ donors without any reported history of cardiovascular disease. The specimens were either fresh frozen at − 80 °C or fixed for 48 h in 2% zinc-paraformaldehyde at room temperature, embedded in paraffin for histology study.

For gene expression analyses, specimens were treated with Trizol (Qiagen) and lysed with a tissue homogenizer (ProScientific, Oxford, MS, USA). RNA isolation was performed using the RNeasy MiniKit (Qiagen, Hilden, Germany) according to the manufacturer’s instructions. Concentration of RNA was determined with a Nanodrop 2000 (Thermo Fisher Scientific, nc., Waltham, MA, USA). cDNA was generated using the High-Capacity RNA-to-cDNA Kit (Thermo Fisher). RT-qPCRs with TaqMan probes (Thermo Fisher) were applied to determine the changes in gene expression.

### Human carotid atherosclerotic plaque single-cell sequencing and data analysis

Human carotid arteries were obtained from patients undergoing carotid endarterectomy or open repair in our Department of Vascular and Endovascular Surgery (Munich Vascular Biobank) as previously published [24].

Single-cell RNA sequencing and analysis were performed as described [24]. The related Microarray data are available from Gene Expression Omnibus data sets (accession number GSE247238).

### Isolation and culture of primary human monocytes

Primary monocytes were isolated from whole blood of healthy individuals obtained from the blood bank of Karolinska University Hospital according to a standard protocol. Briefly, whole blood was diluted 1:1 with phosphate-buffered saline and layered on top of Ficoll-Premium (GE Healthcare) gradient to separate monocytes/lymphocytes from red blood cells and neutrophils by centrifugation (4000* g*, 30 min). The intermediate fraction, containing monocytes and lymphocytes, was collected and residual red blood cells were lysed using ACK (Ammonium-chloride-potassium) lysis buffer. To increase the purity of monocytes, a hyperosmotic Percoll (GE Healthcare) solution was used with subsequent centrifugation (5800 g, 15 min). Monocytes were cultured in Roswell Park Memorial Institute medium (RPMI 1640, Invitrogen), supplemented with 5% fetal bovine serum and 0.1% Penicillin–Streptomycin, differentiated with macrophage colony-stimulating factor (M-CSF, 100 ng/mL) treatment for 7 days, and transformed to macrophages on day 3 with M-CSF and fresh media.

### THP1 cell culture

Human monocytic THP-1 cells were maintained in culture in RPMI culture medium containing 10% of heat inactivated fetal bovine serum and supplemented with 1% l-glutamine 1% penicillin–streptomycin. To transform THP1 monocytes to macrophages, 100 ng/ml PMA (phorbol 12-myristate-13-acetate) was added to the medium for 24 h and one day without PMA prior to the treatment.

### Immunofluorescence staining

Monocytes plus M-CSF were seeded on coverslips (7 days), after treatment they have been fixed with 4% paraformaldehyde for 2 h. The cells were washed three times with PBS and permeabilized in 0.1% Saponin (20 min). After blocking with 2.5% BSA, the cells were incubated overnight with primary antibodies followed by staining with Alexa-dye labeled secondary antibodies. Images were obtained using LSM 700 Confocal Laser Scanning Microscope (Carl Zeiss). Images were processed either in Zen software (Carl Zeiss) or via Fiji (ImageJ).

### Preparation of oxidized low-density lipoprotein (oxLDL)

Lipoproteins used for foam cell formation were isolated through sequential ultracentrifugation from human plasma obtained from the blood bank. Briefly, plasma was ultracentrifuged for > 22 h at 285,000 g at 4 °C. The intermediate fraction containing low‐density lipoprotein (LDL) and high‐density lipoprotein (HDL) was collected. The density of the LDL/HDL fraction was adjusted to 1.063 g/ml with potassium bromide (Sigma‐Aldrich) and ultracentrifuged as described above. The upper fraction with LDL was collected and desalted using a PD‐10 column (GE Healthcare). LDL was oxidized overnight at 37 °C using 20 μM copper sulphate [CuSO_4_] (Merck), and the reaction was stopped using 1 mM EDTA (Sigma‐Aldrich).

To produce FITC-labelled oxLDL, Fluorescein Isothiocyanate (FITC, Sigma-Aldrich) was dissolved in DMSO and added to LDL (10 µM). Then, incubated in dark under rotation for 4 h at 4 °C. The remaining FITC was removed by PD10 column (GE Healthcare).

### Foam cell formation

Human monocyte differentiated macrophages were exposed to 25 µM FITC-labelled oxLDL for 24, 48 and 72 h, foam cell formation was confirmed by fluorescence microscopy and observation of lipid droplets of the macrophages.

### Live cell imaging for apoptosis

Human monocyte differentiated macrophages were seeded in a 12- or 24-well plate. To assess the effect of PIEZO1 on apoptosis, CellEvent Caspase-3/7 Green Detection Reagent (ThermoFisher) was used according to the manufacturer’s instructions and the images were obtained with the use of live cell imaging IncuCyte HD system (Essen BioScience). Cultures were maintained at 37 °C in an XL-3 incubation chamber (Carl Zeiss) and run-in quadruplicates.

### Phagocytosis assay

Human monocyte differentiated macrophages were seeded on glass coverslips in 24 well plate and stimulated with 50 µM Yoda1 (Tocris Bioscience, UK) for 1 h. Without changing the medium, pHrodo^™^ Green Zymosan Bioparticles™ were added for an additional 90 min followed by addition of LysoTracker Red (1 µM) before terminating the experiment. Cells were fixed with 4% paraformaldehyde and mounted on the slide for imaging.

### PIEZO1 silencing using siRNA

To knockdown *PIEZO1* gene expression in monocyte differentiated macrophages, siRNA targeting human *PIEZO1* or scrambled (negative control) was purchased from Dharmacon (L-020870-03-0005, D-001810-10-05) [25]. 20 nM siRNA were transfected using Lipofectamine RNAiMax (Invitrogen) for 3 days.

### Calcium influx measurement

Human monocyte differentiated macrophages were seeded on glass-bottom dishes (ibidi) and treated with Yoda1 (50 µM) or DMSO as solvent control for 2 h. Thereafter, they were stained with Fluo4-AM (1 µM, Life Technologies) for 30 min before starting imaging on 63 × oil immersion objective of LSM 700 Confocal Laser Scanning Microscope (Carl Zeiss).

### Live cell mitochondrial fragmentation, mtROS, and cytosolic ROS assays

Mitochondria fragmentation, mitochondrial reactive oxygen species (mtROS), and cytosolic ROS were assessed on cultured human monocyte differentiated macrophages seeded on glass-bottom dishes (ibidi GMbH, Gewerbehof Gräfelfing, Germany). Yoda1 (50 µM) or DMSO solvent control was added to medium for 2 h, then 30 min before imaging either MitoTracker Deep Red (1 µM, ThermoFisher) or MitoSOX (1 µM, ThermoFisher) or H2DCFDA (10 µM, ThermoFisher) was added to the medium. Images were obtained using 63 × oil immersion objective of LSM 700 Confocal Laser Scanning Microscope (Carl Zeiss).

### Extracellular ROS assay

Monocyte differentiated macrophages were seeded in 96 well plate and treated immediately with Lucigenin-enhanced chemiluminescence (500 µM) and Yoda1 (50 µM) or DMSO solvent control. Luminescence values were detected by Glomax multi detection system (Promega).

### RNA isolation and processing of macrophages

RNA was extracted from cells using RNeasy Mini Kit according to a modified manufacturer’s protocol (Qiagen). Reverse transcription was carried out using SuperScript III Reverse Transcriptase (Thermo Fisher Scientific) according to the manufacturer’s instructions. RT-qPCR was carried out using TaqMan gene expression assays (Thermo Fisher Scientific) and StepOne plus Real-Time PCR System (Thermo Fisher Scientific). Relative gene expression was determined using the ^ΔΔ^CT method (online methods).

### Western blot

Monocyte-derived macrophages were lysed in a buffer containing 50 mmol/L HEPES (pH 7.4), 1% Triton X‐100 (v/v), complete protease inhibitor cocktail, and PhosSTOP phosphatase inhibitor cocktail (both from Roche Diagnostics). Equal amounts of protein were loaded to SDS‐PAGE using 4–20% Criterion XT Bis‐Tris precast gels (Bio‐Rad, Hercules, CA) followed by transfer onto polyvinylidene fluoride membrane (Immobilon‐P; Millipore). Incubation with primary antibodies was performed in the same buffer overnight at 4 °C. Peroxidase‐conjugated IgG was used as secondary antibody. The SuperSignal West Dura Extended Duration Substrate from Thermo Fisher Scientific was used for enhanced chemiluminescence detection. The signals were visualized and evaluated on a ChemiDoc Touch Imaging System (Bio‐Rad).

### Animal gain and loss function experiments

Male *ApoE*^*−/−*^ mice (6–8 weeks old) and high fat diet were purchased from Nanjing Qingzilan Technology Co., Ltd (Nanjing, China). All mice were fed on chow diet until they were 16 weeks old and then changed to a high fat diet (1.5% cholesterol, 10% lard, 4% milk powder, 0.5% sodium cholate) for 4 weeks before and during the 4-week treatment period (online Fig. 4A). The mice were randomly divided into three treatment groups (n = 6 per group): vehicle (saline) control; PIEZO1 antagonist (GsMTx4); and PIEZO1 agonist (Yoda1). GsMTx4 (270 µg/kg, ab141871, Abcam) [26] and Yoda1 (70 μg/kg, SML1558-25MG, Sigma-Aldrich) [27] diluted in saline and 200 μl of each treatment were injected intraperitoneally twice a week for 4 weeks. Tissues were collected 3 days after the last intraperitoneal injection. All mice were housed in a specific pathogen-free environment on a 12 h light/dark cycle at 25 °C with free access to high fat diet and water. The animal protocol was approved by the Harbin Medical University Ethics Committee (13766830089). The use and care of the animals conformed to the US National Institutes of Health Guide for Care and Use of Laboratory Animals.

### Mouse blood pressure measurement

The CODA^™^ mouse/rat tail-cuff system (USA) was used to noninvasively monitor the mice’s blood pressure before and after injection every week. The blood pressure was measured in a quiet standardized animal room on a heating pad at 30 to 35 °C.

### Mouse serum lipid profiling

Mouse peripheral blood was collected. After standing at room temperature for 2 h, the blood was centrifuged under the condition of 4 °C, 3000 r/min for 15 min. Serum was separated for the determination of total cholesterol, low-density lipoprotein cholesterol and triglycerides with the kits (A111-2; A110-1-1, A113-1-1, Nanjing Jiancheng Bioengineering Institute, China).

### Evaluation of mouse peritoneal macrophage oxLDL uptake

High fat-diet fed *ApoE*^−/−^ mice were euthanized with CO_2_ and immersed in 75% alcohol for 5 min. Peritoneal macrophages were harvested via peritoneal lavage with 5 ml cold phosphate-buffered saline (PBS). The cells were cultured in 35-mm petri dishes in RPMI-1640 medium containing 15% FBS. Adherent peritoneal macrophages were used for subsequent experiments. The cells were transformed into foam cells by incubating with 25 μg/ml oxLDL in serum-free RPMI-1640 medium for 24 h. Cells were fixed with 4% paraformaldehyde. The staining was measured according to the manufacturer’s instructions of the Oil Red O staining kit (Solarbio) and were observed under light microscope (Olympus) with quantification using Image-Pro Plus 6.0 software (Media Cybernetics).

### Histology and immunohistochemistry

For immunohistochemistry, consecutive tissue sections of 3 µm thickness were incubated for 1 h at room temperature with primary antibodies detailed in Table S1. Secondary antibody and DAB-mediated detection reagents were provided with the DAKO REAL Detection Kit Rabbit/Mouse. Nuclear counterstaining was performed with hematoxylin staining.

Mouse atherosclerotic lesions were evaluated using H&E and Oil Red O (Solarbio kit) staining with cryosections of the aortic roots. Masson’s trichrome staining was performed to assess the collagen contents in atherosclerotic plaques.

Images were obtained using an Olympus microscope. The positive area relative to the plaque area was analyzed using a computer-assisted color image analysis system (Image-Pro Plus, version 6.0, Media Cybernetics, Inc.)

Immunofluorescence staining was performed on cryosections of murine aortic roots. The sections were incubated with anti-PIEZO1 antibody, anti-CD206 antibody, and anti-iNOS antibody at 4 °C overnight. Rinsed sections were then incubated with FITC conjugated anti-F4/80 PE antibody for 1 h at room temperature in the dark and 4′,6-diamidino- 2-phenylindole (DAPI) for additional 2 min. The fluorescent images were obtained using an Olympus BX53F microscope (Olympus). All parameters were measured by computer-assisted color image analysis (Image-Pro Plus, version 6.0, Media Cybernetics, Inc.).

### Terminal deoxynucleotidyl transferase dUTP nick end labeling (TUNEL) assay

Cell apoptosis was determined by In Situ Cell Death Detection Kit (Roche, 11684817910). Aortic root sections were incubated with TUNEL reaction mixture and anti-F4/80-PE. Followed by DAPI incubation. Sections were analyzed with a fluorescence microscope (Olympus BX53F) and the percentage of TUNEL positive cells was analyzing using an ImageJ software.

### Statistics

All data is presented as the mean ± standard deviation. The difference among groups was analyzed via Students t-test or one-way analysis of variance (ANOVA) followed by the Tukey's multiple comparisons test. Differences with *P* < 0.05 were statistically significant (*P < 0.05, **P ≤ 0.01, ***P ≤ 0.001, ****P ≤ 0.0001). The statistical analyses were performed using GraphPad Prism 8.0.

## Results

### Increased PIEZO1 expression in human and murine atherosclerotic plaques

**Fig. 1:**
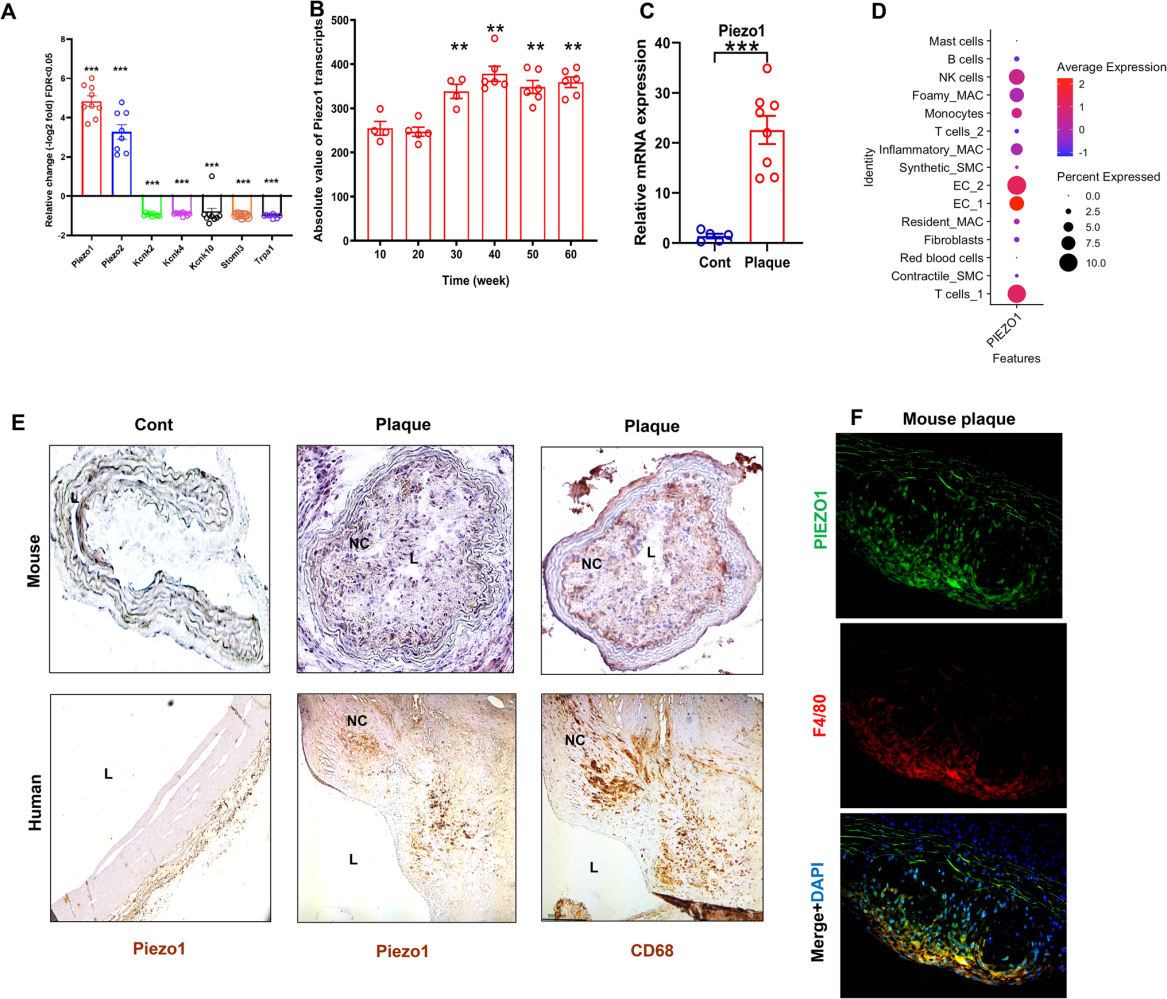
Advanced human and mouse atherosclerotic plaques have higher PIEZO1 expression. Microarray data reveals **A** upregulated *Piezo1* transcripts in carotid plaques in comparison to non-atherosclerotic controls upon ligation in *ApoE*^*−/−*^ mice (*n* = 9, unpaired t test, ***P < 0.001), **B** gradual upregulation of *Piezo1* transcripts in aortic atherosclerotic plaque of high fat diet fed *ApoE*^*−/−*^ mice (*n* = 4–6, One-way ANOVA, **P < 0.01), **C** *PIEZO1* mRNA expression is higher in atherosclerotic human plaque compared to control carotid artery (n = 5–8, unpaired t test, ***P = 0.0001), **D** single-cell sequencing dot plot showing the expression pattern of PIEZO1 in identified cell clusters. **E** Representative immunohistochemistry staining depicting higher co-expression of PIEZO1 and the macrophage marker CD68 in the human plaque and mice plaque as compared to control aorta. The lumen of aorta is denoted by “L”, **F** immunofluorescence of mouse aortic plaque shows accumulation of mouse PIEZO1 (green) in the macrophages (F4/80, red) in the plaque area. Piezo type mechanosensitive ion channels (Piezo), two-pore domain K + channels (Kcnk), Stomatin‐like protein 3 (Stoml3), Transient receptor potential cation channel subfamily A member 1(Trpa1)

Carotid plaque whole transcriptome array of *ApoE*^−/−^ mice that had undergone carotid ligation revealed 2 upregulated genes Piezo-type mechanosensitive ion channel component 1 and 2 (*Piezo1, Piezo2*)*,* 5 down-regulated ion channel genes, Potassium Two Pore Domain Channel Subfamily K Member 2,4,10(*Kcnk2,4,10*), Stomatin-Like Protein 3(*Stoml3*) and Transient Receptor Potential Cation Channel Subfamily A Member 1(*Trpa1*), among which *Piezo1* as the most prominent ion channel transcript upregulated in advanced carotid atherosclerotic lesions compared to uninjured control vessels (Fig. 1A). Analysis of the Gene Expression Omnibus (GEO) dataset GSE43292 which included 32 patients with atheroma plaque and adjacent carotid tissue revealed significantly higher PIEZO1 transcripts in carotid artery plaques compared with macroscopically intact tissue (Supplementary material online, Fig. S1A) [28].

Time course transcriptome array of mouse aortic lesion confirmed that *Piezo1* expression increased with atherosclerosis progression (Fig. 1B). More importantly, increased *PIEZO1* mRNA expression was also found in advanced human carotid plaques compared with control arterial tissue (Fig. 1C, (Supplementary material online, Fig. S1B)). When we further analyzed our human carotid plaque single cell sequencing data, we found it is mostly expressed in endothelial and immune cells (Fig. 1D) but downregulated in inflammatory and foamy macrophages. Immunohistochemistry of mouse and human atherosclerosis demonstrated higher levels of PIEZO1 protein in plaques, together with higher expression of CD68 positive monocytes and macrophage-like cells when compared with control artery (Fig. 1E). PIEZO1 staining was also found to co-localize with the macrophage marker F4/80 in aortic plaques of mice (Fig. 1F). Taken together, these observations showed increased expression of PIEZO1 in atherosclerotic plaques associated with accumulation of macrophages.

### PIEZO1 activation induces apoptosis in foam cells and subsequent phagocytosis activity of macrophages

**Fig. 2:**
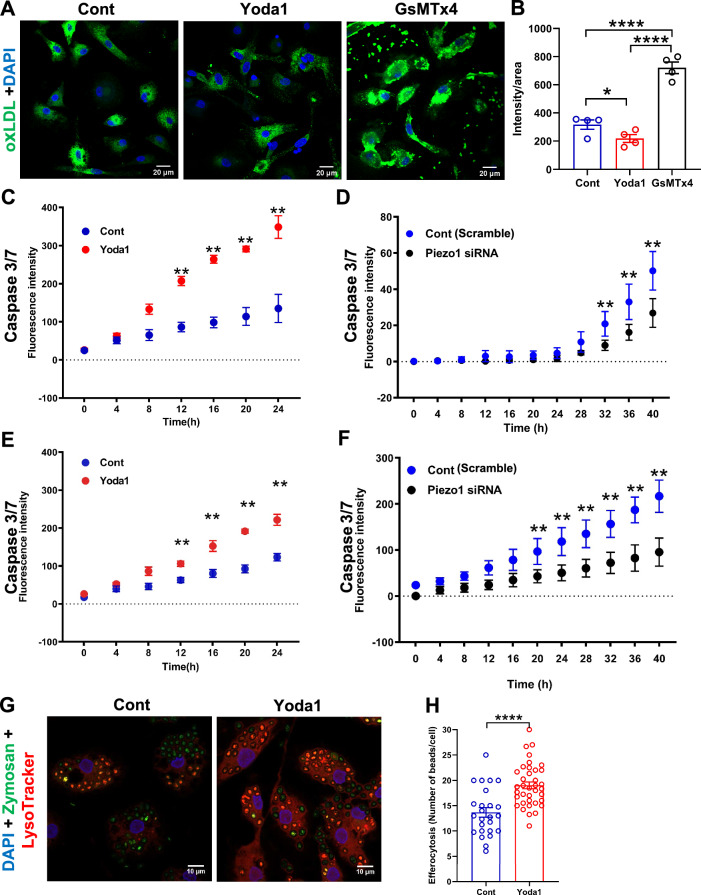
PIEZO1 regulates oxLDL uptake, apoptosis and phagocytosis by macrophages. **A**, **B** Modulating PIEZO1 with its agonist Yoda1 reduced FITC-labeled oxLDL uptake, whereas PIEZO1 antagonist GsMTx4 enhanced the FITC-labeled oxLDL uptake (n = 4, One-way ANOVA, *P < 0.05, ****P < 0.0001). **C** PIEZO1 activation promotes apoptosis of monocyte-derived foam cells, indicated by Caspase 3/7 activity (n = 3–4, paired test, **P < 0.01). **D** *PIEZO1* downregulation using siRNA retards apoptosis of foam cells compared to scrambled siRNA (n = 6, paired test, **P < 0.01). **E**, **F** PIEZO1 activation by Yoda1 promotes apoptosis of macrophages with lower intensity than foam cells (n = 3–4, paired test, **P < 0.01), *PIEZO1* downregulation using siRNA retards apoptosis of macrophages cells compared to scrambled siRNA, though to lesser extent than in foam cells (n = 6). **G**, **H** Yoda1 increased phagocytosis of zymosan beads (green) and their targeting to the lysosomes (red) (n = 25–35 cells from 4 independent experiments, unpaired t test, ****P < 0.0001)

Activation of PIEZO1 by pretreatment with Yoda1 the specific activator of PIEZO1 [29] for 2 h resulted in reduced oxLDL uptake by macrophages, while PIEZO1 inhibitor GsMTx4 (500 nM) pretreatment (1 h) significantly increased oxLDL uptake (Fig. 2A, B). Intriguingly, macrophages that accumulate lipids upon treatment with oxLDL for 72 h, showed reduced PIEZO1 mRNA and protein levels compared to the untreated controls (Fig. 1D, Supplementary material online, Fig. S1C, D). PIEZO1 activation by Yoda1 enhanced foam cell apoptosis while PIEZO1 silencing suppressed foam cell apoptosis (Fig. 2C, D). Of note, PIEZO1 activation in oxLDL-loaded foam cells promoted higher apoptosis levels compared with non oxLDL loaded macrophages (Fig. 2E, F), demonstrating a pivotal role of PIEZO1 on macrophage and foam cell viability. Consistently, in human monocyte THP1 derived healthy macrophages PIEZO1 activation decreased expression of the pro-apoptotic genes *CASPASE 3* and *CASPASE 9* by 20–40%, accompanied by reduction of *FAS* expression by 60%. Further, PIEZO1 activation increased gene expression of the anti-inflammatory and anti-apoptotic Fas apoptotic inhibitory molecule 3 (*FAIM3*) by fourfold (Supplementary material online, Fig. S1E–H). Enhanced phagocytosis of zymosan bioparticles was observed in macrophages upon PIEZO1 activation by Yoda1 for 2 h, with most of the engulfed particles targeted to lysosomes as reflected by co-localization with lysosomal marker lysotracker red (Fig. 2G, H).

### PIEZO1 activation alters mitochondrial dynamics and reactive oxygen species (ROS) production in macrophages

**Fig. 3:**
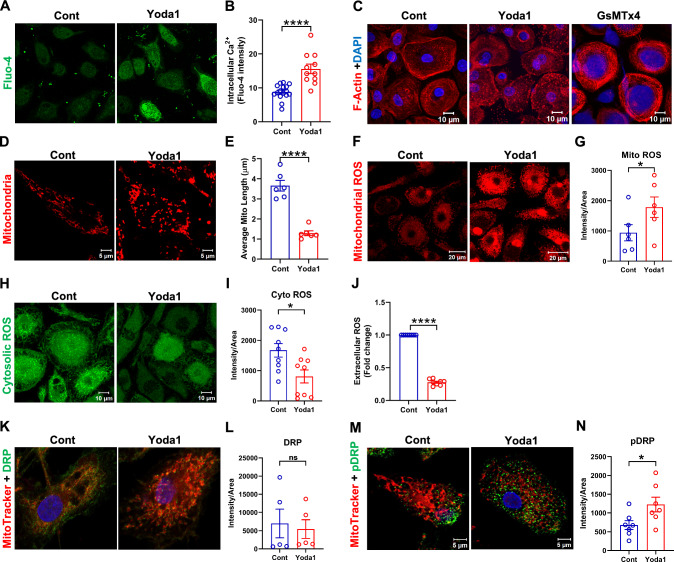
PIEZO1 activation promotes mitochondrial fission by generating mitochondrial ROS and phosphorylated DRP1. **A**, **B** Fluorescence analysis demonstrating activation of PIEZO1 by its selective activator Yoda1 for 2 h in monocyte-differentiated macrophages results in higher cytosolic Ca^2+^ content in monocyte-derived macrophages (n = 3, each containing 9–15 cells, unpaired t-test, ****P < 0.0001). **C** Representative confocal images of Phalloidin (red) stained human macrophages. Exposing macrophages to PIEZO1 agonist Yoda1 affects F-actin rearrangement in macrophages. Control and GsMTx4 treated macrophages show concentric submembranous actin filaments. **D**, **E** PIEZO1 activation enhances mitochondrial fission (fragmentation) reflected by lower mitochondrial length (MitoTracker Red staining, n = 6). Piezo1 activation in macrophages **F**, **G** increases mitochondrial ROS levels (MitoSOX, red, n = 6), **H**, **I** decreases cytosolic ROS levels (H2DCFDA, green, n = 9), J reduces extracellular ROS (lucigenin-enhanced chemiluminescence, n = 8), **K**, **L** increases mitochondrial association of DRP1 without influencing total DRP1 expression levels (n = 5), **M**, **N** enhances pDRP1 levels (n = 7). Paired t test, *P < 0.05, ***P < 0.0001

PIEZO1 activation using Yoda1 for 2 h induced a robust increase in intracellular Ca^2+^ levels (Fig. 3A, B), confirming the presence of functional PIEZO1 in human macrophages. Since F-actin directly drives macrophage phagocytosis and movement [30], we assessed F-actin (re)arrangement upon modulation of PIEZO1 activity. PIEZO1 activation dramatically induced macrophage F-actin depolymerization, while inhibition led to the formation of more cortical F-actin (Fig. 3C), suggesting that PIEZO1 activation triggers dynamic remodeling of the actin cytoskeleton which is required for cell polarity, cell migration, vesicle trafficking, phagocytosis, and lysosomal activity.

Although PIEZO1 is expressed constitutively in human primary monocyte-derived macrophages, we observed increased expression of PIEZO1 upon activation by Yoda1 (Supplementary material online, Fig. S2A). A small proportion of PIEZO1 was also found to be localized to the mitochondria (Fig. S2B). Microarray data of mouse carotid plaque showed significant changes in expression of genes involved in mitochondrial fission, such as BCL2 Associated X(*Bax*), Hexokinase 2(*Hk2*), Spermatogenesis Associated 18(*Spata18*), Mitochondrial Elongation Factor 1(*Mief1*) and parkinson protein 2 (*Park2*) (Supplementary material online, Fig. S2C). Interestingly, PIEZO1 activation also induced transient mitochondrial fission in macrophages, as indicated by a reduction in the average mitochondrial length (Fig. 3D, E). Since calcium signaling and mitochondrial fission–fusion cycle has been associated with mitochondrial ROS production [31–33], we explored the impact of PIEZO1 activation on macrophage ROS production. PIEZO1 activation significantly increased mitochondrial ROS (mtROS) production (Fig. 3F and G) and decreased both cytosolic and extracellular ROS (Fig. 3H–J).

Dynamin-related protein 1 (DRP1) phosphorylation is one of the critical upstream stimuli of mitochondrial fission and ROS production [34]. Yoda1 notably increased both association of DRP1 to mitochondria (Fig. 3K, L) and enhanced phosphorylated DRP1 (pDRP1) levels in macrophages, without significant change in total DRP1 levels (Fig. 3M, N). We also observed that PIEZO1 activation led to an increase in *NOS2* expression in THP1-macrophages (Supplementary material online, Fig. S2D), which is associated with mitochondrial depolarization and metabolic rewiring [35, 36]. Moreover, PIEZO1 activation in THP1-macrophages significantly increased the mRNA levels of anti-inflammatory molecules *TNFAIP6*, *PPARG* and *IL10*, while inflammatory molecule TNF level remained comparable (Supplementary material online, Fig. S3A–D).

### PIEZO1 activation suppressed lipid accumulation in tissue resident macrophages

To further observe the therapeutic potential of PIEZO1 activation, ApoE^−/−^ mice were treated with PIEZO1 specific agonist Yoda1 [37, 38], PIEZO1 inhibitor GsMTx4 [39, 40] or saline as vehicle control twice weekly for 4 weeks (Supplementary material online, Fig. S4A). Neither Yoda1 nor GsMTx4 treatment affected body weight, systolic blood pressure, serum concentration of total cholesterol, LDL, triglycerides, and total macrophage count in the plaques of the mice (Supplementary material online, Fig. S4B–G). Interestingly, ex vivo analysis of the peritoneal macrophages from Yoda1 treated mice demonstrated significantly decreased lipid accumulation, while macrophages from GsMTx4 treated mice showed significantly higher lipid content (Supplementary material online, Fig. S5A). This indicated that PEIZO1 activation tends to decrease lipid accumulation in macrophages.

### PIEZO1 activation attenuates mouse atherosclerosis formation and influence plaque phenotype

**Fig. 4:**
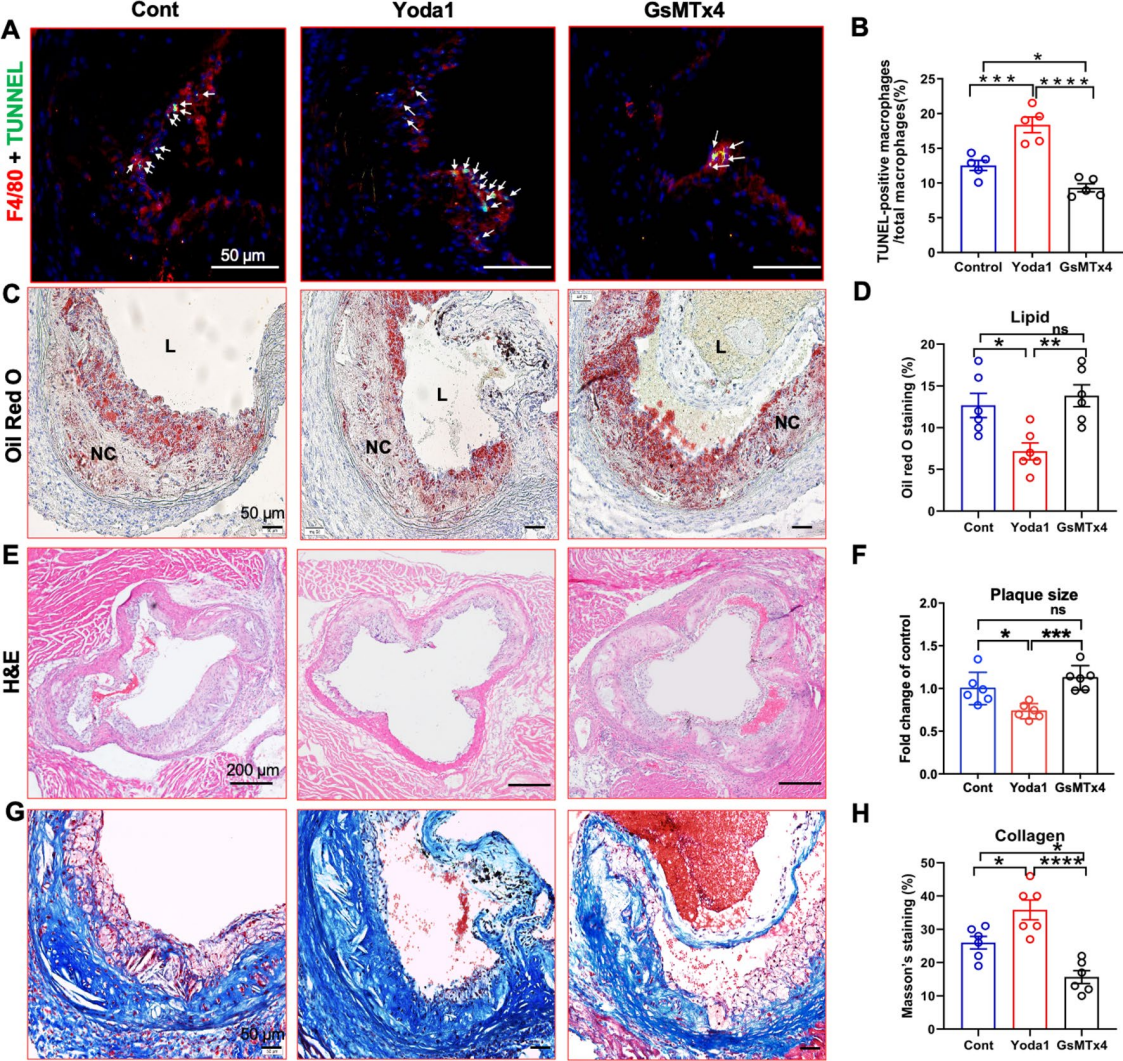
PIEZO1 activation attenuates mouse atherosclerosis formation and influence plaque phenotype. **A**, **B** TUNNEL staining is used to measure apoptosis levels (green) in macrophages in the aortic plaque of high fat diet fed *ApoE*^*−/−*^ mice (n = 5) treated with Yoda1 or GsMTx4. **C**–**H** Representative images and quantification of aorta lesion from high fat diet fed *ApoE*^*−/−*^ mice (n = 6) treated with Yoda1 or GsMTx4 compared to untreated control stained with, **C**, **D** oil red O to evaluate lipid accumulation, **E**, **F** hematoxylin and Eosin to measure plaque area, **G**, **H** Masson’s Trichome to assess collagen deposition. One way ANOVA with Tukey's multiple comparisons test. *P < 0.05, **P < 0.01, ***P < 0.001, ***P < 0.001, ****P < 0.0001

To ascertain the role of PEIZO1 in atherogenesis, examined the changes in aortic plaques in response to treatment with PIEZO1 modulators. Consistent with the in vitro and ex vivo data described above, PIEZO1 activation enhanced apoptosis in mouse aortic plaques, revealed by larger number of TUNNEL positive macrophages/foam cells in the aortic plaque (Fig. 4A, B). PIEZO1 inhibition decreased apoptosis of macrophages/foam cells. Oil red O staining demonstrated that Yoda1 treated mice accumulated lower lipid content in their aortic lesions (Fig. 4C, D). Quantification of the area of hematoxylin and eosin-stained aortic plaques revealed remarkably suppressed plaque development in Yoda1 treated mice compared with untreated control mice (Fig. 4E, F). Further, Yoda1 treatment increased collagen content in aortic plaques, indicating increased plaque stability after PIEZO1 activation (Fig. 4G and H).

**Fig. 5:**
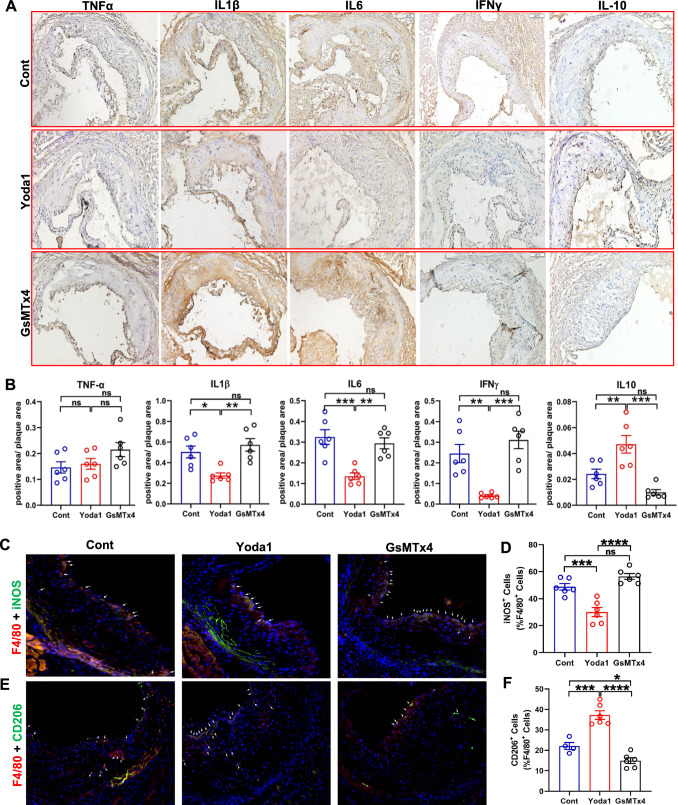
PIEZO1 activation decreases inflammatory milieu in mice aortic plaques. **A** Representative images of aorta and **B** quantification of relative protein expression of pro and anti-inflammatory cytokines measured using immunohistochemistry of aorta from *ApoE*^−/−^ mice fed on high fat diet demonstrating distribution and expression levels of TNFα, IL1β, IL6, IFNγ and IL10 (n = 6). Negative control is presented (online Fig. 6). **C, D** Immunofluorescence of plaque lesions double-stained for F4/80 (red) and iNOS (green), and its quantification and **E, F** F4/80 (red) CD206 (green) and quantification of corresponding double-positive cells in mice plaques (Arrows represent typical examples, n = 4–6). One-way ANOVA with Turkey's multiple comparisons test, *P < 0.05, **P < 0.01, ***P < 0.001, ****P < 0.0001

The Yoda1 treated mouse aortas showed reduction of proinflammatory proteins IL1β, IL6, and IFNγ, but not TNFα, with concomitant increase in anti-inflammatory protein IL10 (Fig. 5A, B; Supplementary material online, Fig. S5B). Piezo1 inhibition by GsMTx4 did not show any noticeable differences in tissue cytokine levels. Notably, aortic plaques of Yoda1 treated mice displayed significantly decreased number of iNOS^+^ pro-inflammatory M1 macrophages, and significantly increased number of CD206^+^ M2 macrophages, indicating that PIEZO1 activation drives the macrophages towards a more protective anti-inflammatory phenotype (Fig. 5C–F).

## Discussion

Ion channels play a crucial role in regulating macrophage function [41, 42]. Here, we present the first evidence that the calcium channel PIEZO1 regulates macrophage functions critical for progression of atherosclerosis. Among the various mechanosensitive ion channels that we analyzed in advanced murine carotid plaques, *Piezo1* showed the highest increase, indicating a prominent role in atherogenesis. Our in vitro*, *ex vivo and in vivo functional studies confirmed the expression of PIEZO1 channel in macrophages and revealed its modulatory action.

PIEZO1 expression was found to be the highest among all known mechanosensitive channels in macrophages, supporting our observations [14]. Activation of PIEZO1 with the specific agonist Yoda1 significantly impacted oxLDL accumulation, either by reducing oxLDL uptake [43] or enhancing lipid metabolism within macrophages. This reduction in oxLDL accumulation points to a pivotal role of PIEZO1 in foam cell formation. Interestingly, mitochondrial fragmentation enhances lipid oxidation capacity and preference for lipids as a fuel source [44, 45], this fragmentation affects carnitine O‐palmitoyltransferase 1 (CPT1)'s sensitivity to its allosteric inhibitor, malonyl-CoA, providing a mechanism by which mitochondrial structure regulates fatty acid oxidation [44]. Our observations suggest that increased mitochondrial fragmentation and reduced lipid accumulation upon PIEZO1 activation in macrophages are linked. This implies that PIEZO1 activation alters mitochondrial architecture and impacts the metabolic profile of macrophages. A significant decrease in PIEZO1 levels in macrophages and foam cells may exacerbate atherosclerosis, prolonging foam cell presence within lesions and intensifying the inflammatory microenvironment. Our human single-cell sequencing results confirm this effect, promoting monocytic infiltration and differentiation within plaques, thus increasing overall PIEZO1 levels.

The balance between formation and clearance of foam cells is crucial for plaque stability. Efficient clearance of apoptotic foam cells contributes to a stable plaque phenotype, whereas their accumulation within the plaque leads to engorgement of necrotic core, inflammation [46], and formation of rupture prone plaque [47]. Our results show that activating PIEZO1 with Yoda1 enhances apoptosis of oxLDL-laden foam cells, reducing their accumulation. Additionally, PIEZO1 activation boosts macrophage phagocytic capacity, enhancing the clearance of apoptotic cells [15, 48]. The result of this study shows that oxLDL accumulation in macrophages and PIEZO1 have synergistic effect on apoptosis. Based on our observation, we hypothesis that Yoda1 increases phagocytosis as a downstream action, triggered by calcium influx-induced remodeling of the F-actin cytoskeleton. Interestingly, activation of PIEZO1 in control macrophages (those not treated with oxLDL) did not induce significant apoptosis unlike the effect observed in ox-LDL-treated foam cells. This difference could be attributed to the observed reduction in pro-apoptotic genes *Caspase3, Caspase9*, coupled with an increase in anti-apoptotic *FIAM3* expression, as observed in THP1-macrophages upon PIEZO1 activation. Therefore, PIEZO1 induces apoptosis in foam cells and macrophages with varying intensity, possibly due to differences in the expression of pro- and anti-apoptotic genes upon activation.

This study confirms that macrophage mobility and motility highly depend on Ca^2+^ influx and mtROS production [32, 33, 49]. The altered macrophage mitochondrial morphology upon PIEZO1 activation could be due to mtROS elevation and *NOS2* expression, which has been reported to trigger mtROS production and mitochondrial depolarization [35, 36]. We found that Yoda1 induced mitochondrial DRP1 phosphorylation, which could trigger mtROS production, then inducing mitochondrial fragmentation [50]. While our data suggest a potential link between PIEZO1-mediated mitochondrial fission via DRP1 and the subsequent increase in mtROS, leading to enhanced phagocytosis, it's important to note that these findings need to be confirmed through further investigation.

**Fig. 6:**
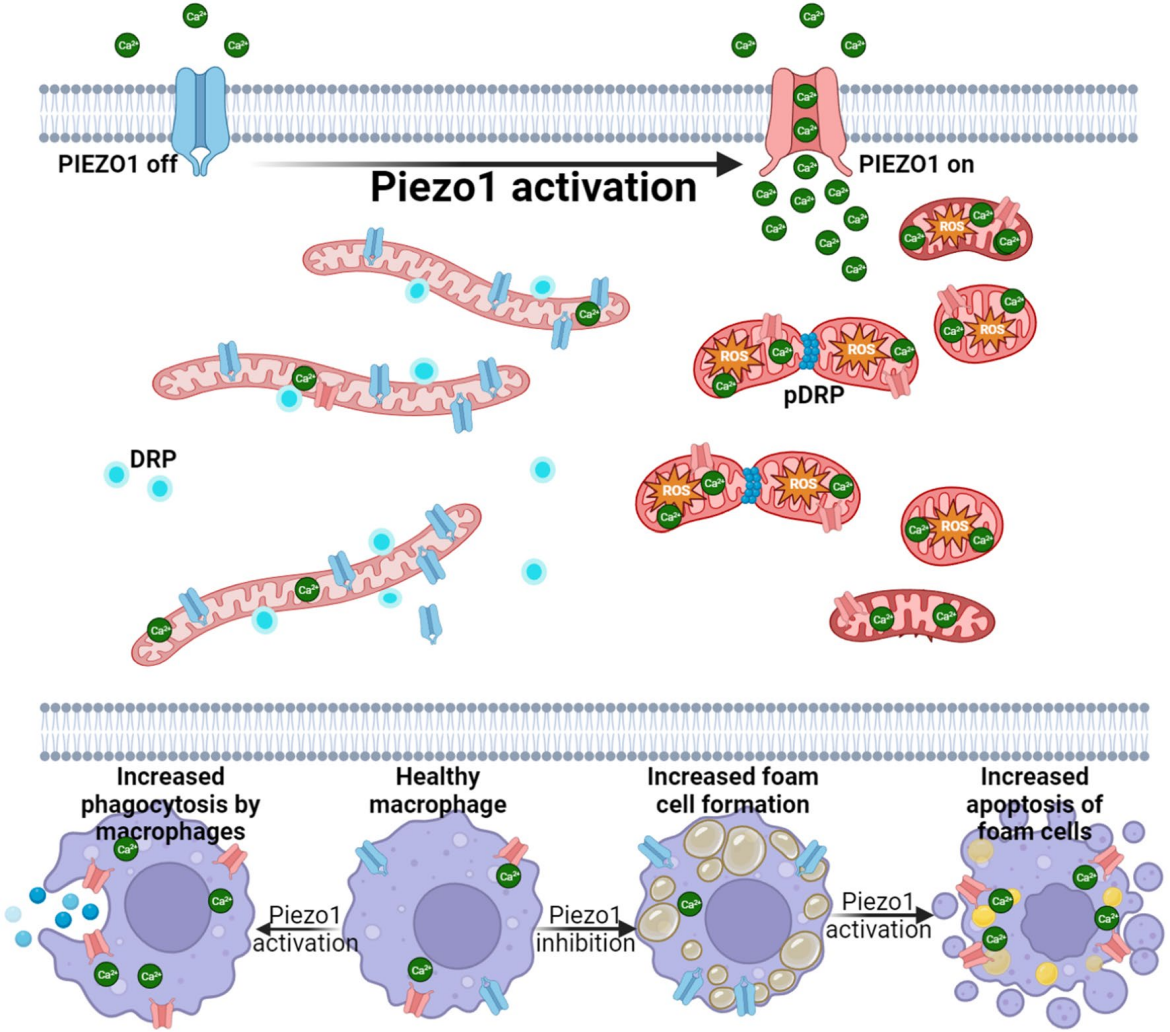
Proposed mechanism of PIEZO1 activation reducing oxLDL accumulation in macrophages, while enhancing phagocytosis and apoptosis of foam cells. Activation of PIEZO1, a mechanosensitive ion channel with its specific activator, Yoda1, enhances Ca^2+^ influx that results in mitochondrial fission via phosphorylated DRP1 activity and increased mtROS. PIEZO1 activation augments phagocytosis due to calcium induced membrane and actin remodeling in macrophages. Moreover, macrophage PIEZO1 inhibition results in lipid accumulation and foam cell formation, whereas activation of PIEZO1 promotes apoptosis of foam cells

The protective role of PIEZO1 activation was strongly reflected in the animal studies. Treatment of *ApoE*^−/−^ mice with PIEZO1 agonist Yoda1 attenuated tissue resident macrophage lipid uptake, indicating the inhibition of foam cell formation. Consistent with in vitro and ex vivo results, Yoda1 decreased mouse aortic lesion size, lipid content, without any systemic effects on serum lipid profile. Moreover, the plaques of Yoda1 treated mice showed collagen enrichment as well as an increased anti-inflammatory signature and decreased pro-inflammatory cytokines IL1$\beta$, IL6 and IFN$\gamma$, which have been reported to enhance plaque stability [51]. The refinement of macrophages with anti-inflammatory phenotype in mice subjected to PIEZO1-activation highlights the importance of PIEZO1, systemic Yoda1 administration potentially exert effects on other vascular cells such as endothelial cells and smooth muscles. In this study we focused on the effect of pharmaceutical activation of PIEZO1 in macrophages in context of atherosclerosis, though our observation could reflect cumulative effects of systemic Yoda1 administration. Moreover, in vitro experiment upon Piezo1 silencing and activation may further reveal the undelying molecular mechanisms, which we haven’t achieved in the present study, but can be elucidated in our future experiments. The present study highlights PIEZO1 as a potential novel target for atherosclerosis intervention. This is achieved by regulation of foam cell apoptosis and phagocytosis capacity macrophages, and mitigating inflammation, which are pivotal processes in atherosclerosis progression (Fig. 6).

### Supplementary Information

Below is the link to the electronic supplementary material.

Supplementary file1 (PDF 2112 KB)

## Data Availability

The human datasets generated and analysed during the current study are accessible through GEO Series accession number GSE247238. One mouse transcriptome database is accessible through GEO Series accession number GSE38574. Other data that support the findings of this study are available from the corresponding author upon reasonable request.
