# Supplementary material for: PIEZO1 targeting in macrophages boosts phagocytic activity and foam cell apoptosis in atherosclerosis
Source: Cell Mol Life Sci. 2024 Aug 6;81(1):331. doi: 10.1007/s00018-024-05372-3 (PMC11335255; doi:10.1007/s00018-024-05372-3)
Supplement: Supplementary file 1 — Supplementary file1 (PDF 2112 KB) [file 18_2024_5372_MOESM1_ESM.pdf]

PIEZO1 targeting in macrophages boosts phagocytic activity and foam cell apoptosis in atherosclerosis

Shirin Pourteymour[1,2*#], Jingxue Fan[3*], Rakesh Kumar Majhi[4], Shuyuan Guo[3], Xin Sun[5], Zhen Huang[3], Ying Liu[3], Hanna Winter[6], Alexandra Backlund[1], Nikolaos-Taxiarchis Skenteris[1,7], Ekaterina Chernogubova[1], Olivera Werngren[1], Zhaolong Li[6], Josefin Skogsberg[8], Yuhuang Li[7], Ljubica Matic[7], Ulf Hedin[7], Lars Maegdefessel[1,6], Ewa Ehrenborg[1], Ye Tian[3#], Hong Jin[1,7#]

[1]Department of Medicine (Solna), Karolinska Institutet, Karolinska, Stockholm, Sweden

[2]Department of Nutrition, Institute of Basic Medical Sciences, Faculty of Medicine, University of Oslo Norway.

[3]Department of Cardiology, The First Affiliated Hospital, Cardiovascular Institute, Harbin Medical University, Harbin, PR China.

[4]Department of Microbiology, Tumor and Cell Biology, Division of Clinical Microbiology, Karolinska Institutet, Sweden

[5]Department of Cardiology, Shenzhen Cardiovascular Minimally Invasive Medical Engineering Technology Research and Development Center, Shenzhen People's Hospital, Shenzhen, PR China

[6]Department of Vascular and Endovascular Surgery, Technical University Munich, Munich, Germany

[7]Department of Molecular Medicine and Surgery, Karolinska Institutet, Stockholm, Sweden

[8]Department of Medical Biochemistry and Biophysics, Karolinska Institutet, Stockholm, Sweden

* These authors contributed equally to the manuscript

[#]Correspondence to:

Hong Jin MD, PhD, Department of Molecular Medicine and Surgery, Karolinska Institute, Stockholm, Sweden

Email: hong.jin@ki.se

Ye Tian MD, PhD, Department of Cardiology, The First Affiliated Hospital, Cardiovascular Institute, Harbin Medical University, Harbin, PR China

Email: yetian@ems.hrbmu.edu.cn

Shirin Pourteymour, PhD, Department of Nutrition, Institute of Basic Medical Sciences, Faculty of Medicine, University of Oslo, Blindern, PO Box 1046, 0317, Oslo, Norway

Email: shirin.pourteymour@medisin.uio.no

Short title: PIEZO1 regulates macrophage function in atherosclerosis

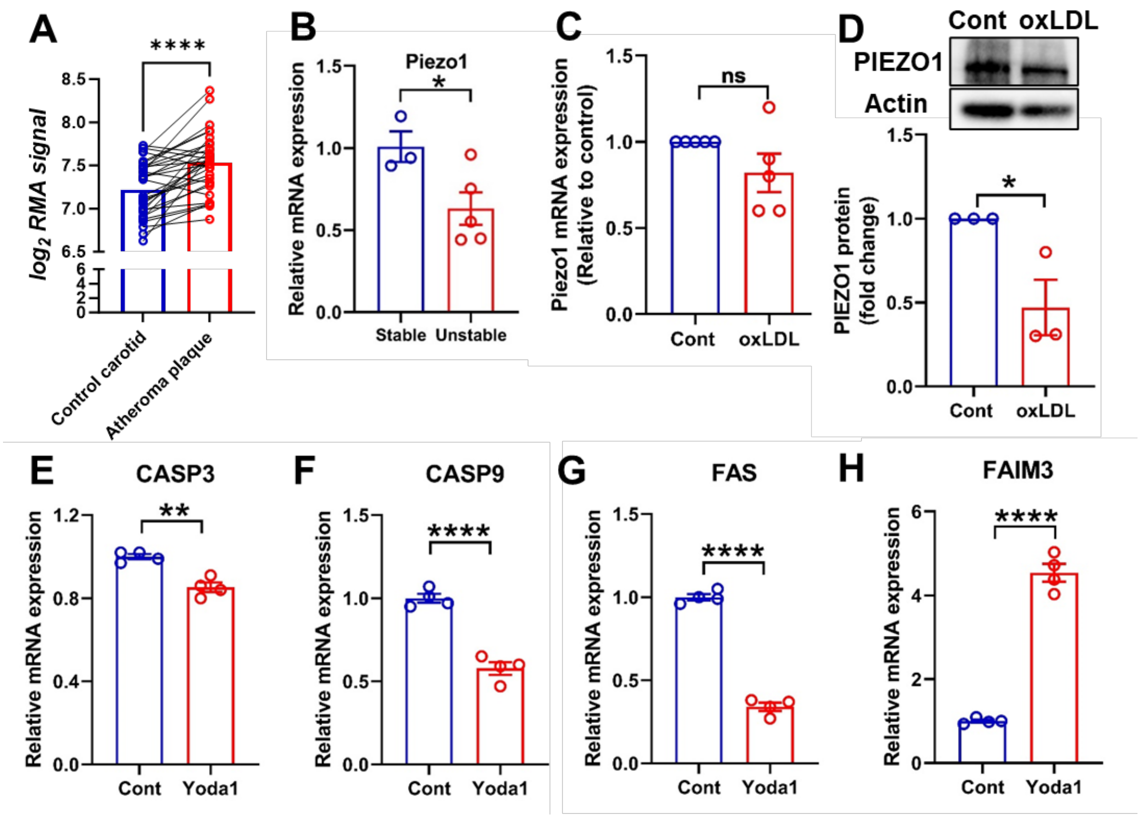

Supplementary Fig. 1 PIEZO1 regulation in macrophages. (A) *PIEZO1* mRNA in human carotid plaque and adjacent healthy carotid artery (n=39, paired t-test, *****P*<0.00001). **(B)** *PIEZO1* mRNA expression is higher in stable plaque compared with unstable plaque (n=3-5). **(C, D)** Exposing monocyte-derived macrophages to 25 µg/ml oxLDL for 72 h did not affect the *PIEZO1* mRNA level, but partly decreased PIEZO1 protein level (n=3-5, paired test, **P*<0.05). Expression levels of genes involved in apoptosis **(E)** *CASP3*, **(F)** *CASP9*, **(G)** *FAS*, **(H)** *FAIM3* are regulated in THP1 cells exposed to Yoda1 24 h compared to only vehicle treated controls (n=4), Unpaired t-test, **P*<0.05, ***P*<0.01, *****P*<0.0001).

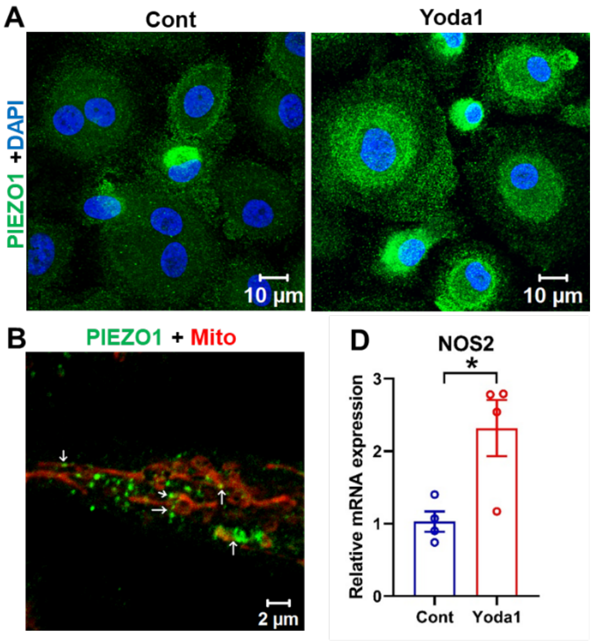

C

Gene ID	Fold Change	ANOVA p-value
Bax	1.44	0.00054
Bbc3	1.02	0.417331
Dnm1l	-1.1	0.170422
Hk2	1.64	0.007834
Mief2	1.1	0.455483
Pld6	1.09	0.846421
Mtfr1	1	0.522191
Opa1	1.17	0.631207
Plekhf1	1.04	0.604341
Spata18	-3.2	0.000782
Mief1	1.2	0.03088
Park2	-1.5	0.001816

Supplementary Fig. 2 PIEZO1 affects macrophages phenotype and function. (A) Yoda1 increases PIEZO1 expression or re-localization in macrophages. **(B)** PIEZO1 (green) is distributed at the plasma membrane, cytoplasm and is also associated with the mitochondria (red) in macrophages. Arrows indicate regions of co-localization. **(C)** Microarray data of mouse carotid plaque showed significant changes in genes involved in mitochondrial fission. **(D)** *NOS2* mRNA levels is reduced in THP1 cells in response to Yoda1 exposure for 24 h compared to only vehicle treated controls (n=4, unpaired t-test, **P*<0.05)

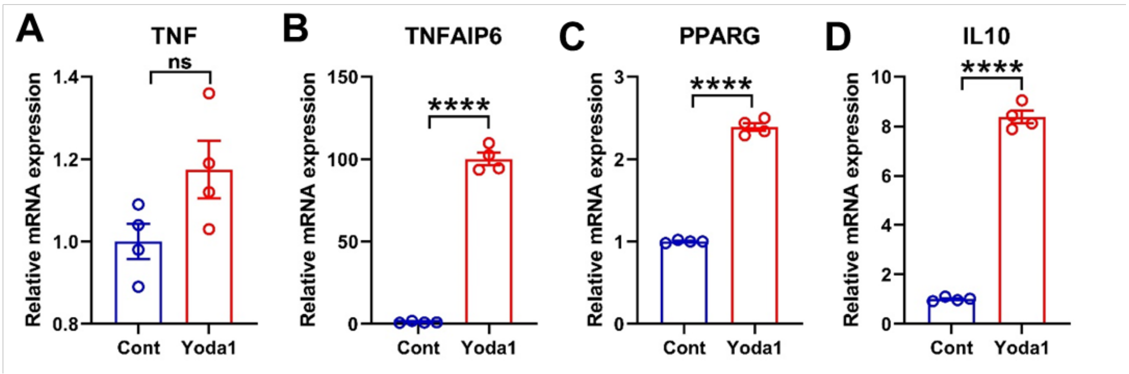

Supplementary Fig.3. PIEZO1 activation promotes anti-inflammatory cytokines in macrophages. Expression of inflammatory genes markers (A) TNF, (B) TNFAIP6, (C) PPARG, (D) IL10 in THP1 cells in response to Yoda1 exposure for 24 h compared to only vehicle treated controls (n=4, unpaired t-test, ****P<0.0001).

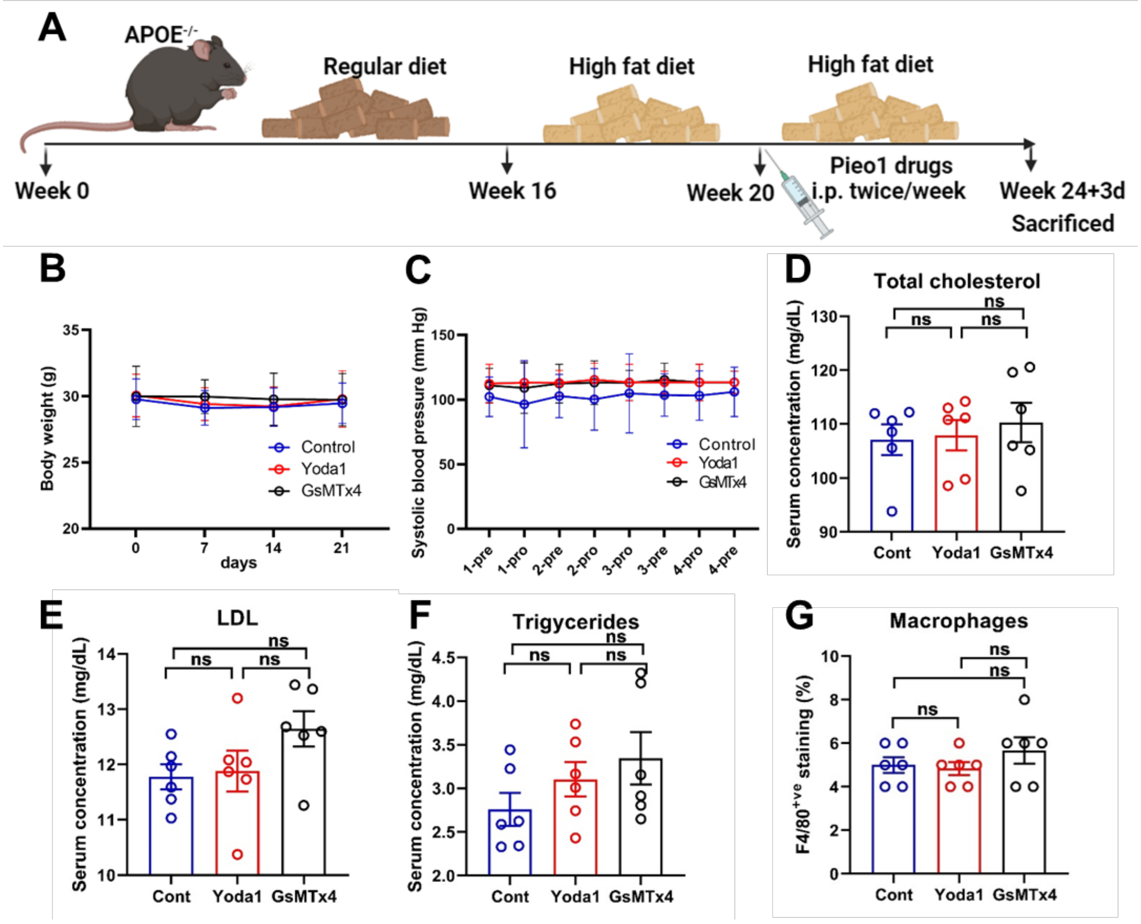

Supplementary Fig. 4. Peritoneal injection of PIEZO1 regulator did not change mice body phenotype. (A) Schematic representation of experimental plan shows 8w old *ApoE*$^{-/-}$ mice fed with a high fat diet for 4w, then administered intraperitoneal injection of Piezo1 activator Yoda1, inhibitor GsMTx4 or saline solvent as control for 4w (twice a week), followed by sacrifice and tissue collection. Following parameters remained unchanged after treatment of *ApoE*$^{-/-}$ mice with Yoda1, GsMTx-4 or saline control: (**B)** animal's body weight, (**C)** systolic blood pressure, **(D)** total serum cholesterol, **(E)** serum LDL, **(F)** serum triglyceride, and **(G)** number of macrophages in the plaque.

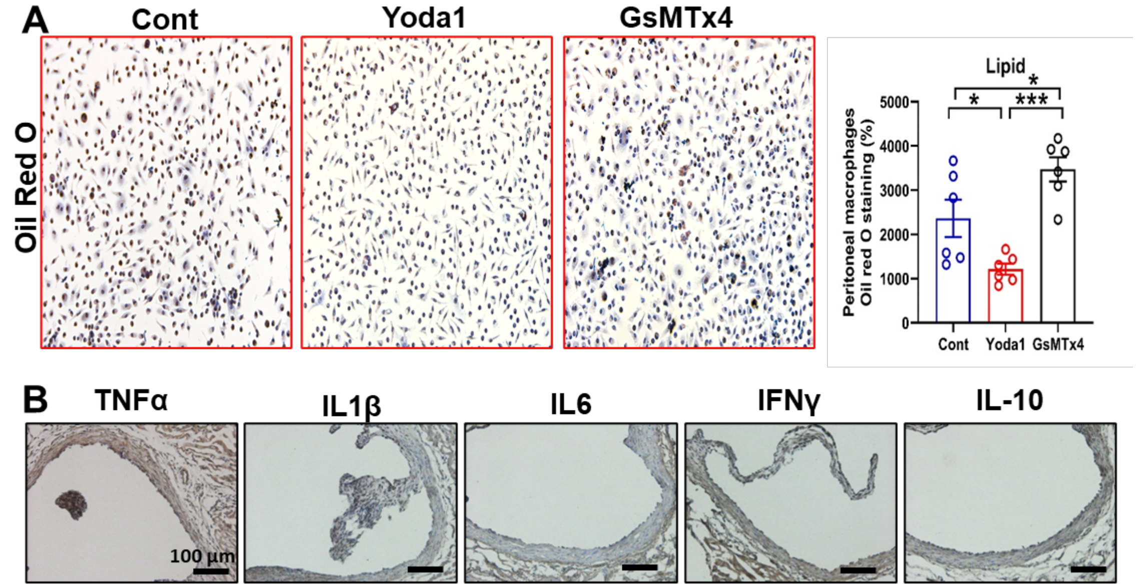

Supplementary Fig. 5. Piezo1 decreases lipid uptake by macrophages. (A) Oil red O staining of peritoneal macrophages from high fat diet fed ApoE$^{-/-}$ mice depicting levels of lipid uptake upon Piezo1 modulation. **(B).** Representative images of aorta stained with the same protocol as in Fig 5A (depicting expression profiles of TNFα, IL1β, IL6, IFNγ and IL10) in the aorta from *ApoE*$^{-/-}$ mice fed on high fat diet, with the corresponding primary antibodies omitted during immunohistochemistry staining.

Supplementary table1

Materials are used for human studies.

Phalloidin Conjugated to Alexa Flour 488	ThermoFisher	A12379
Phalloidin Conjugated to Alexa Flour 555	ThermoFisher	A30106
FLuo-4 AM	ThermoFisher	F14201
PIEZO1 antibody	abcam	Ab128245
DRP1 antibody	abcam	Ab56788
pDRP1 antibody	Sigma-Aldrich	SAB4301399
MitoSOX	ThermoFisher	M36008
MtroTracker deep red	ThermoFisher	M22426
Yoda1	Millipore Sigma	SML 1558
GsMTx-4	abcam	Ab141871
pHrodo™ Green Zymosan Bioparticles™	ThermoFisher	P35365
Lucigenin-enhanced chemiluminescence	Millipore Sigma	M8010
LysoTracker red	ThermoFisher	L7528
CellEvent Caspase-3/7 Green Detection Reagent	ThermoFisher	C10423
H2DCFDA	ThermoFisher	D399
RPLP0	Thermo Fisher	Hs999999902_m1
CASP3		Hs00234387_m1
CASP9		Hs00609647_m1
FAS		Hs00236330_m1
FAIM3 Hs00193770_m1		Hs00193770_m1
TNF Hs01113624_g1		Hs01113624_g1
TNFAIP6 Hs00200180_m1		Hs00200180_m1
PPARG Hs01115513_m1		Hs01115513_m1
NOS2 Hs01075529_m1		Hs01075529_m1

Supplementary table2

Materials are used for animal studies.

oxLDL	Yiyuan Biotechnologies	Guangzhou,China
TNF-α antibody	Bioss Inc. (Beijing, China)	bs-2081R
IL-10 antibody	Bioss Inc. (Beijing, China)	bs-0698R
IL-6 antibody	Bioss Inc. (Beijing, China)	bsm-10807m
IFN-γ antibody	Bioss Inc. (Beijing, China)	bs0480R
iNOS antibody	Bioss Inc. (Beijing, China)	bs0162R
PIEZO1 antibody	Abcam	ab128245
CD206 antibody	Santa Cruz Biotechnology	sc-34577
IL-1β (3A6) antibody	Cell Signaling	12242S
F4/80 antibody	GeneTex	GTX101895
PE-tagged anti-F4/80 antibody	eBioscience	12-4801-80
